# Editorial: Regulatory mechanisms of gene expression, chromatin structure and nuclear dynamics in gametogenesis

**DOI:** 10.3389/fcell.2022.995650

**Published:** 2022-08-31

**Authors:** Soichiro Yamanaka, Satoshi H. Namekawa, Kei-ichiro Ishiguro

**Affiliations:** ^1^ Department of Biological Sciences, Graduate School of Science, The University of Tokyo, Tokyo, Japan; ^2^ Department of Microbiology and Molecular Genetics, University of California, Davis, Davis, CA, United States; ^3^ Department of Chromosome Biology, Institute of Molecular Embryology and Genetics (IMEG), Kumamoto University, Kumamoto, Japan

**Keywords:** Gametogenesis, gene expression, epigenetics, chromatin, sex determination

## Introduction

Primordial germ cells differentiate into gametes through complex processes over a long period of time. Along the way, environmental signals are integrated into the chromatin state and epigenome, leading to molecular and cellular phenotypes. As a result, a diverse set of genomic and epigenomic information is passed on to the next generation. Despite a common principle of sexual dimorphism in which two types of gametes are produced and passed on to the next generation, the repertoire of genes involved and their functions are extremely diverse among species.

This article Research Topic covers papers on sexual reproduction in mammals as well as non-mammalian model organisms, such as medaka and *Daphnia*. These article Research Topic should facilitate a better understanding of the molecular pathways associated with gametogenesis.

## H3K4me3 imprinting

Genomic imprinting is regulated by differential epigenetic states between the parental alleles and controls development in both eutherian and marsupial mammals (Renfree et al., 2009). Ishihara et al. identified novel paternally imprinted genes by re-analyzing public sequence data sets of sperm and early embryos published in previous mouse studies. They found that these novel paternally-expressed genes in zygotes are associated with H3K4me3 in the paternal genome in sperm and zygotes at the pronuclear stage 3 (PN3), thus identifying an epigenetic inheritance mechanism that is distinct from DNA methylation- and H3K27me3-based imprinting (Inoue et al., 2017). The article discusses the transgenerational effect of sperm H3K4me3 on zygotic genome activation in a contest of histone-to-protamine exchange (Yamaguchi et al., 2018) and their paternal allele-specific expression during preimplantation development.

## piRNA

PIWI family proteins maintain genome integrity by repressing transposable elements. Its target specificity is conferred via interacting small RNA, called Piwi-interacting (piRNA) (Onishi et al., 2021). In mice, depletion of any member of PIWI family proteins results in defects in spermatogenesis, showing their crucial role in male fertility. Two major types of piRNAs have been identified in testicular germ cells: pre-pachytene piRNA and pachytene piRNA, which are expressed in distinct developmental stages. Pre-pachytene piRNA expression starts in prospermatogonia (also known as gonocytes), prior to the pachytene stage of meiotic prophase I. On the other hand, pachytene piRNA expression begins after the pachytene stage of meiotic prophase I (Czech et al., 2018). piRNAs are processed from their precursor transcripts, which are derived from specific genomic loci called piRNA clusters. In this research paper, Kawase and Ichiyanagi identified three types of piRNA clusters: prospermatogonial, early, and late clusters, using piRNA datasets from purified stage-specific germ cells, proposing a revised model of a cluster- or sequence-dependent mechanism for loading onto PIWI-like proteins.

## Mini-puberty

Shima provides a mini-review on the latest knowledge related to the role of mini-puberty in spermatogonial stem cell (SSC) formation. This phenomenon is known as temporal activation of the hypothalamic-pituitary-gonadal axis during the neonatal period (Kuiri-Hänninen et al., 2014). Mini-puberty coincides with prospermatogonia-SSC transformation in the seminiferous tubules in humans. Studies of human cryptorchid patients raised the hypothesis that mini-puberty plays a role in SSC formation (Hadziselimovic and Hoecht, 2008). However, this mini-review points out contradictory observations in human and mouse studies for the hypothesis.

## Medaka

Kikuchi and Tanaka provide a perspective article proposing the existence of two functional modules that regulate meiosis and folliculogenesis in the teleost fish medaka. The topic focuses on the gene regulatory mechanism of the germ cell-fate decision and how meiosis and other programs related to gametogenesis are integrated. Currently, the mechanism remains largely elusive. Medaka is a model organism in which germline stem cells continuously produce eggs and sperm in mature gonads. By contrast, in mammals, early stages of oogenesis occur in the fetus, and spermatogenesis occurs after adolescence. *Foxl3* has been identified as a germ-cell intrinsic Sperm-Egg fate determinant in medaka ovaries (Nishimura et al., 2015). *Foxl3* functions as a switch gene acting upstream of *rec8a*, which promotes female-specific meiosis, and of *fbxo-47*, which regulates folliculogenesis (Kikuchi et al., 2019; Kikuchi et al., 2020). The authors propose that these two pathways serve as “functional modules” by which expression of a group of genes is coordinated in the same developmental process in germ cells.

## Daphnia

Most organisms that create offspring through sexual reproduction have two sexes. The systems of sex determination are broadly categorized into two types: genetic and environmental. In most animals, sex is genetically determined by specific genetic elements, such as *Sry* (Gubbay et al., 1990; Sinclair et al., 1990), whereas in taxa such as fish and reptiles, sex is determined by environmental cues (Korpelainen, 1990). In *Daphnia*, eggs are developed to become males in response to external stimuli, such as crowding and lack of food. Otherwise, only females are produced by parthenogenesis in healthy conditions. In this review by Kato and Watanabe, they summarize the current knowledge in *Daphnia*, specifically how the expression of the *Dsx1* gene, which has a central role in sex determination, is regulated during the late oogenesis in the mother’s body through multi-layered mechanisms, including transcription factors, RNA-binding proteins, and lncRNAs. From these data, they provide a model of how the upregulation and maintenance of robust *Dsx1* expression are interconnected to trigger masculinization.

## Conclusion

Due to the limited amounts of materials at each stage during gametogenesis, it often proves challenging to elucidate molecular events with classical biochemical approaches. However, over the past decade, with the tour de force of two biotechnologies (high-throughput DNA sequencing technologies and genome-editing tools), substantial progress has been made in understanding the mechanisms underpinning gene regulation during gametogenesis. Further technical innovation would shed light on hitherto unknown mechanisms of gene expression, chromatin structure, and nuclear dynamics in gametogenesis of diverse organisms.

## References

[B1] Czech B. Munafò M. Ciabrelli F. Eastwood E. L. Fabry M. H. Kneuss E. (2018). piRNA-guided genome defense: From biogenesis to silencing. Annu. Rev. Genet. 52, 131–157. 10.1146/annurev-genet-120417-031441 30476449 PMC10784713

[B2] Gubbay J. Collignon J. Koopman P. Capel B. Economou A. Münsterberg A. (1990). A gene mapping to the sex-determining region of the mouse Y chromosome is a member of a novel family of embryonically expressed genes. Nature 346, 245–250. 10.1038/346245a0 2374589

[B3] Hadziselimovic F. Hoecht B. (2008). Testicular histology related to fertility outcome and postpubertal hormone status in cryptorchidism. Klin. Padiatr. 220, 302–307. 10.1055/s-2007-993194 18401814

[B4] Inoue A. Jiang L. Lu F. Suzuki T. Zhang Y. (2017). Maternal H3K27me3 controls DNA methylation-independent imprinting. Nature 547, 419–424. 10.1038/nature23262 28723896 PMC9674007

[B5] Kikuchi M. Nishimura T. Ishishita S. Matsuda Y. Tanaka M. (2020). foxl3, a sexual switch in germ cells, initiates two independent molecular pathways for commitment to oogenesis in medaka. Proc. Natl. Acad. Sci. U. S. A. 117, 12174–12181. 10.1073/pnas.1918556117 32409601 PMC7275758

[B6] Kikuchi M. Nishimura T. Saito D. Shigenobu S. Takada R. Gutierrez-Triana J. A. (2019). Novel components of germline sex determination acting downstream of Foxl3 in medaka. Dev. Biol. 445, 80–89. 10.1016/j.ydbio.2018.10.019 30392839

[B7] Korpelainen H. (1990). Sex ratios and conditions required for environmental sex determination in animals. Biol. Rev. Camb. Philos. Soc. 65, 147–184. 10.1111/j.1469-185x.1990.tb01187.x 2190635

[B8] Kuiri-Hänninen T. Sankilampi U. Dunkel L. (2014). Activation of the hypothalamic-pituitary-gonadal axis in infancy: Minipuberty. Horm. Res. Paediatr. 82, 73–80. 10.1159/000362414 25012863

[B9] Nishimura T. Sato T. Yamamoto Y. Watakabe I. Ohkawa Y. Suyama M. (2015). Sex determination. foxl3 is a germ cell-intrinsic factor involved in sperm-egg fate decision in medaka. Science 349, 328–331. 10.1126/science.aaa2657 26067255

[B10] Onishi R. Yamanaka S. Siomi M. C. (2021). piRNA- and siRNA-mediated transcriptional repression in Drosophila, mice, and yeast: new insights and biodiversity. EMBO Rep. 22, e53062. 10.15252/embr.202153062 34347367 PMC8490990

[B11] Renfree M. B. Hore T. A. Shaw G. Marshall Graves J. A. Pask A. J. (2009). Evolution of genomic imprinting: Insights from marsupials and monotremes. Annu. Rev. Genomics Hum. Genet. 10, 241–262. 10.1146/annurev-genom-082908-150026 19630559

[B12] Sinclair A. H. Berta P. Palmer M. S. Hawkins J. R. Griffiths B. L. Smith M. J. (1990). A gene from the human sex-determining region encodes a protein with homology to a conserved DNA-binding motif. Nature 346, 240–244. 10.1038/346240a0 1695712

[B13] Yamaguchi K. Hada M. Fukuda Y. Inoue E. Makino Y. Katou Y. (2018). Re-Evaluating the localization of sperm-retained histones revealed the modification-dependent accumulation in specific genome regions. Cell Rep. 23, 3920–3932. 10.1016/j.celrep.2018.05.094 29949774

